# The many facets of disseminated parenchymal brain cysticercosis: A differential diagnosis with important therapeutic implications

**DOI:** 10.1371/journal.pntd.0009883

**Published:** 2021-11-18

**Authors:** Oscar H. Del Brutto, Hector H. Garcia

**Affiliations:** 1 School of Medicine, Universidad Espíritu Santo—Ecuador, Samborondón, Ecuador; 2 Center for Global Health, Department of Microbiology, Universidad Peruana Cayetano Heredia, Lima, Perú; 3 Cysticercosis Unit, Instituto Nacional de Ciencias Neurológicas, Lima, Perú; Federal University of Ceará, Fortaleza, Brazil, BRAZIL

## Abstract

Neurocysticercosis (NCC), the infection of the nervous system by the cystic larvae of *Taenia solium*, is a highly pleomorphic disease because of differences in the number and anatomical location of lesions, the viability of parasites, and the severity of the host immune response. Most patients with parenchymal brain NCC present with few lesions and a relatively benign clinical course, but massive forms of parenchymal NCC can carry a poor prognosis if not well recognized and inappropriately managed. We present the main presentations of massive parenchymal NCC and their differential characteristics.

Infection of the central nervous system by the larval stage of *Taenia solium*—the pork tapeworm—causes neurocysticercosis (NCC), a highly pleomorphic disease [1]. This pleomorphism is partly related to differences in the number and anatomical location of lesions, the viability of parasites, and the severity of the host immune response against the infection. Cysticerci may be located within the brain parenchyma, the subarachnoid space, the ventricular system, the spinal cord, the sellar region, or even the subdural space.

Most patients with parenchymal NCC present with few lesions and a clinical course that is often more benign than that observed in the subarachnoid and ventricular forms of NCC, where a sizable proportion of patients are left with disabling sequelae or may even die as a result of the disease [2,3]. Nevertheless, massive forms of parenchymal NCC require special attention to reduce the risk of complications related to the disease itself or to an inadequate treatment. Here, we present the main presentations of massive parenchymal NCC and their differential characteristics. There is no standardized definition of how many cysts constitute massive NCC. While the term “massive” has usually been applied when there are more than 100 lesions in the brain parenchyma, others have used smaller numbers (50), and there is not a defined cutoff.

## Massive parenchymal brain calcifications

**Fig 1:**
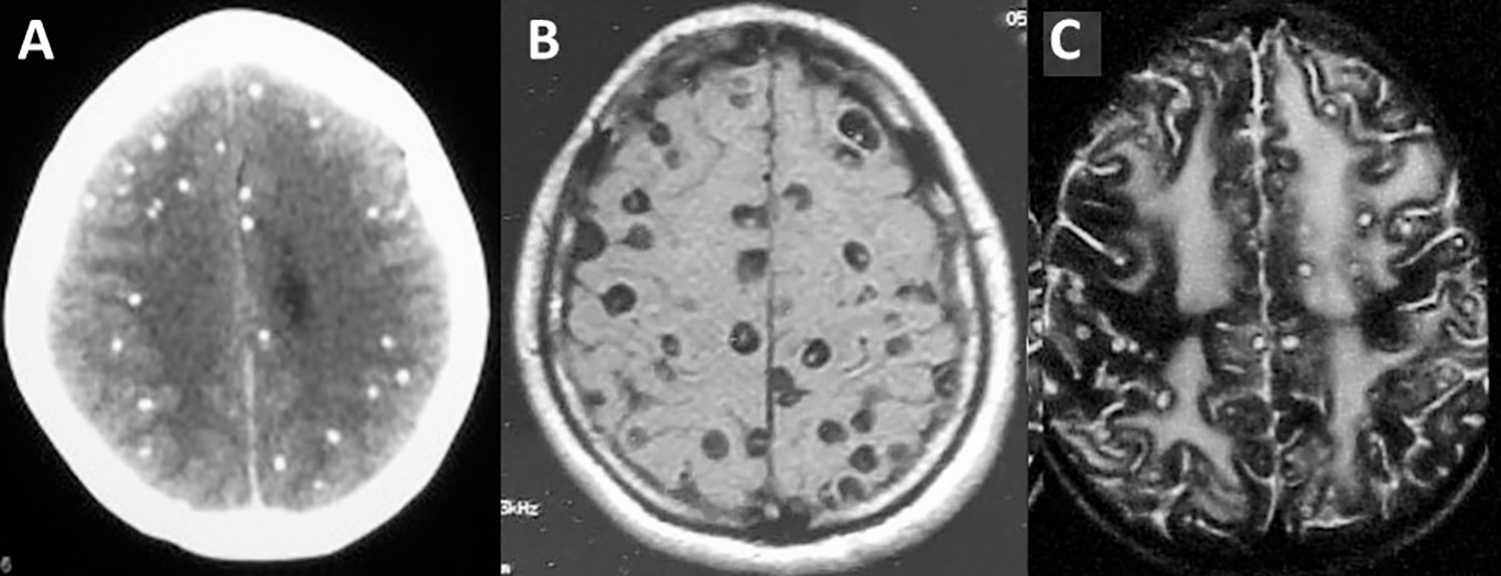
Neuroimaging characteristics of massive parenchymal brain cysticercosis. **(A)** Unenhanced CT scan showing multiple hyperdense parenchymal brain calcifications. **(B)** T1-weighted MRI of a patient with heavy nonencephalitic NCC, showing multiple living brain cysts with an eccentric bright dot representing the scolex. There is no abnormal enhancement or parenchymal brain edema. **(C)** T2-weighted MRI of a patient with cysticercotic encephalitis. Multiple small inflamed cysts can be seen, with marked edema. CT, computerized tomography; NNC, neurocysticercosis.

The most benign of the massive forms of NCC is the presence of many calcifications within the brain parenchyma, which represent the sequelae of a previous infestations in which the host’s immune system was able to overcome the acute infection without major neurological complications (Fig 1A). Computerized tomography (CT) shows typical calcifications of NCC, which are solid, rounded, measure less than 10 mm in diameter, and are most often located in the supratentorial compartment [4]. Using MRI, NCC calcifications are seen as signal void lesions that are more evident in T2-weighted, echo gradient, and susceptibility-weighted sequences. These patients most often present with seizures, a normal neurological examination, and do not require any specific therapy with the exception of anti-seizure medication when indicated. In some cases, however, seizures become refractory due to secondary atrophy/sclerosis of the hippocampus. Surgical removal of the damaged hippocampus may help to control the seizure disorder in these cases [5]. Some other patients develop breakthrough seizures from perilesional edema, which probably occurs as a result of the intermittent exposure of the host’s immune system to cysticercal antigens present in the interior of calcifications due to remodeling mechanisms [6]. Intermittent or even long-term continuous corticosteroid therapy has been used in calcified lesions with edema or enhancement, but there are no studies assessing benefits and risks [6]. The problem here is that the release of trapped antigens may cause an inflammatory reaction associated with abnormal ring-like or nodular enhancement and perilesional edema on neuroimaging studies mimicking a colloidal cysticerci, and some patients—particularly those living in areas where NCC is not endemic—are misdiagnosed as having an active infection and incorrectly received cysticidal drugs [7].

## Heavy nonencephalitic cysticercosis

A second form of presentation of massive parenchymal NCC is the so-called “heavy nonencephalitic cysticercosis,” in which multiple, well-defined, vesicular cysts with little or no associated inflammation or evidence of degeneration are found in the brain parenchyma [8]. These cysts are often 5 to 10 mm in diameter and—in most cases—a brilliant eccentric dot, representing an intact scolex, can be seen within the cysts (Fig 1B). In these cases, it is possible that a state of immune tolerance from the host allows the establishment of the parasites in the central nervous system without evidence of perilesional inflammation or cysts’ degeneration. In the original description, these cases were most often observed among *T*. *solium* carriers, and the coexistence of the adult worm in the intestine and parasitic larvaes in the brain could be related to the pathogenesis of this form of NCC [8]. As expected, many patients with this form of NCC are paucisymptomatic (occasional seizures and mild headache) since brain inflammation is mild or absent; however, it is possible that cognition and other cerebral functions may be affected. Regarding therapy, the use of cysticidal drugs must be decided on an individual basis. While some patients with heavy nonencephalitic cysticercosis have benefited with the use of cysticidal drugs, it is likely that simultaneous destruction of hundreds of cysts will be associated with a severe inflammatory reaction that may even threaten the patient’s life. In these settings, the initial use of a simpler cysticidal treatment scheme (low doses of a single drug) rather than a combination of 2 cysticidal drugs as mentioned in the current United States guidelines [9] seems advisable, together with corticosteroids that must be used before, during, and even after cysticidal drug administration [9]. However, such recommendations are mostly based on anecdotal experience, and further studies are needed to understand the benefits and risks of cysticidal drug therapy in these cases.

## Starry sky presentation

A particular subgroup of heavy nonencephalitic cysticercosis is that described by Wadia and colleagues as “stars on a starry night” [10]. This form is characterized by countless living cysticerci in which cystic components of the parasites are very small and difficult to visualize, and the main finding on CT corresponds to hyperdense scolices that resemble calcifications. The particularity here is the small size of the lesions, which makes it difficult the identification of its cystic nature. All 3 originally described patients also had muscular pseudohypertrophy as a consequence of massive involvement of skeletal muscles. Clinically, these patients developed intractable epilepsy, progressive cognitive decline, and evidence of intracranial hypertension. They were treated with the cysticidal drug praziquantel, and all developed neurological deterioration soon after the onset of therapy and eventually died despite the use of corticosteroids in all cases and suboccipital decompressive craniectomy in 2 of them. While the original report gave no clues on the pathogenetic mechanisms involved in this massive infection by small viable cysts, the homogeneity of the lesions suggests a single infection period with a large egg challenge. Indeed, in one of the patients reported by Wadia and colleagues [10], histopathological study suggested that both cerebral and muscular cysts were about the same age, suggesting a single infection.

Subsequent studies using brain MRI facilitated the recognition of scolices and the cystic component of brain lesions, making it clear that this form of NCC consists on small viable cysticerci [11]. Despite the advantage of MRI in demonstrating viable scolices and intact cysts, the term “starry sky,” as originally conceived, has been frequently misunderstood (mostly confused with calcified infections). Now this naming seems to be used more as a cliché to increase readers’ attention than to define this particular form of massive parenchymal NCC. Interestingly, all cases fulfilling criteria for this rare form of parenchymal NCC have been reported from the Indian subcontinent, although there is no evidence to support that they cannot occur in other settings.

## Cysticercotic encephalitis

The most severe presentation of massive parenchymal NCC is that denominated “cysticercotic encephalitis,” in which hundreds or even thousands of cysticerci are simultaneously inflamed by the attack of the host immune system [12]. It has been speculated that this severe immune reaction occurs in nonpreviously exposed individuals who get infected with a heavy load of *T*. *solium* eggs, and it is also possible that these lesions never became fully established viable cysts before they degenerate, as they were attacked soon after their arrival to the brain parenchyma in an early metacestode stage [13]. This severe form of NCC, which predominantly affects young women, is clinically characterized by acute encephalitis with stupor or coma, seizures, and increased intracranial pressure [12]. On neuroimaging studies, multiple ring or nodular enhancing lesions are disseminated throughout the brain parenchyma, and edema may be multifocal or diffuse (Fig 1C). Diagnosis is difficult on neuroimaging grounds alone since tuberculomas, pyogenic brain abscesses, and even metastatic disease may mimic this form of NCC, and immunological tests (in particular a serum immunoblot for detection of anticysticercal antibodies) are crucial for reaching a definitive diagnosis [14]. Treatment of this rare form of NCC is complicated, and the prognosis is grim. Cysticidal drugs are contraindicated during the acute phase of the disease as their use exacerbates the acute inflammatory reaction. During the acute disease, high doses of corticosteroids, osmotic diuretics, and even decompressive craniotomies are advised to reduce the intracranial hypertension that may cause the death of the affected patient [9]. Cysticidal drugs may be used later, if viable or degenerating lesions persist on follow-up neuroimaging examinations.

## Comment

In summary, there are several well-categorized forms of massive parenchymal NCC. Each of these forms present with particular clinical manifestations, neuroimaging findings, and are probably associated with specific pathogenic mechanisms that must be taken into account in order to reduce misdiagnosis before planning a rational therapeutic approach (Table 1). In addition to the aforementioned well-established forms, some patients present with mixed forms of the disease characterized by multiple parenchymal brain cysticerci in different evolutive stages at the same time [15]. Whether this results from repetitive infections or from an incomplete response of the immune system to a massive cysticercal infection is not known. Massive NCC seems to be less frequently reported in recent years, and it is quite possible that urbanization, improvements in sanitation, increasing commercial pig farms, deworming programs, and other factors have resulted in a decrease in the frequency of this presentation. However, when present, it may be life threatening, and appropriated diagnosis and treatment approaches are needed.

**Table 1:** Characteristics of the different forms of massive parenchymal neurocysticercosis.

Form of the disease	Clinical manifestations	Neuroimaging findings	Treatment*
**Massive parenchymal calcifications (Fig 1A)**	Seizures and headaches	CT: solid, rounded calcifications of less than 10 mm in diameter; MRI: signal void lesions (echo gradient and susceptibility-weighted sequences)	Anti-seizure medication; corticosteroids for patients with breakthrough seizures
**Heavy nonencephalitic cysticercosis (Fig 1B)**	Seizures, headache, and possible cognitive decline. Often associated with systemic cysticercosis (parasitic cysts in muscles, subcutaneous tissues, and other organs, not only invading the central nervous system)	CT and MRI: small cystic lesions showing a brilliant eccentric dot representing the scolex. No edema and no contrast enhancement	Anti-seizure medication; cysticidal drugs may not be used if intracranial hypertension is apparent
**Starry sky presentation**	Intractable epilepsy, progressive cognitive decline, and intracranial hypertension. Often associated with systemic cysticercosis	CT: countless living cysticerci. The cystic component is difficult to visualize, and scolices resemble calcifications; MRI: scolices and cystic components are better defined	Anti-seizure medication; corticosteroids; cysticidal drugs may not be used during the acute phase of the disease
**Cysticercotic encephalitis (Fig 1C)**	Stupor or coma, seizures, and intracranial hypertension	CT and MRI: multiple ring or nodular enhancing lesions disseminated throughout the brain parenchyma; edema may be multifocal or diffuse	Anti-seizure medication; corticosteroids; osmotic diuretics or decompressive craniectomy; cysticidal drugs contraindicated

* Based on expert opinion.

CT, computerized tomography; neurocysticercosis.
